# Draft genome sequences of *Bacillus* and *Pseudomonas* species isolated from *Cannabis sativa* L. and *Chelidonium majus* L.

**DOI:** 10.1128/mra.01462-25

**Published:** 2026-05-18

**Authors:** Laura Amaya-Quiroz, Mohammad Jamil Kaddoura, Mamta Rani, Jamil Samsatly, Hacene Meglouli, Mohamad R. A. Sater, Saji George

**Affiliations:** 1 Department of Food Science and Agricultural Chemistry, McGill University 5620, Montreal, Quebec, Canada; 2 BioSun Solutions, Chambly, Quebec, Canada; 3 Day Zero Diagnostics, Watertown, Massachusetts, USA; University of Manitoba, Winnipeg, Manitoba, Canada

**Keywords:** whole genome sequencing, *Bacillus*, *Pseudomonas*, *Cannabis sativa*, *Chelidonium majus*, bacteria endophytes, plant growth promotion, biocontrol, abiotic stress tolerance

## Abstract

Draft genome sequences of seven *Bacillus* strains isolated from *Chelidonium majus* seeds and six *Pseudomonas* strains isolated from *Cannabis sativa* roots and stems are reported. These genome sequences provide a foundation for functional and comparative genomic analyses to unravel potential traits associated with plant growth promotion and protection.

## ANNOUNCEMENT

Endophytic *Bacillus* and *Pseudomonas* genera have been reported in different plant tissues (1, 2). Several members of these genera possess functions related to plant growth-promoting rhizobacteria (PGPR), such as nutrient solubilization, hormone modulation, and antimicrobial activities (3, 4). Scientific evidence suggests that endophytes are influenced by the host plant’s chemical profile (5). Consequently, medicinal plants exhibiting diverse and potent chemical compounds potentially harbor endophytes with unexplored bioactive properties (6, 7). Endophytic communities of *Cannabis sativa* (cannabis) and *Chelidonium majus* (greater celandine) remain underexplored, particularly in terms of functional properties related to plant growth promotion and disease suppression. Hence, this study reports bacterial endophytes isolated from *C. sativa* and *C. majus*, which were provided by a commercial grower in Quebec, Canada. Plant materials were surface sterilized by immersion in hydrogen peroxide (30% w/w) for 7 min, followed by three 5-min rinses in sterile water, and sterility was confirmed by imprinting onto Luria–Bertani (LB) agar. The plant materials were then aseptically sectioned, incubated in sterile water for 20 min, serially diluted (10^−1^ and 10^−3^), and plated on LB and nutrient agar supplemented with 1% sucrose and 10 µg/mL Benomyl. Colonies were selected based on distinct morphology, followed by repeated subculturing to obtain pure isolates. A single colony from each isolate was inoculated into 15 mL of LB broth and incubated overnight at 35°C with shaking at 180 rpm. Genomic DNA was extracted from pelleted cells using the DNeasy Blood and Tissue Kit (Qiagen, Germany), following the manufacturer’s protocol. Sequenced 16S amplicons produced with universal primers 27F/534R (8) were putatively identified by BLAST. Seven *Bacillus* strains (BS-114, BS-115, BS-116, BS-118, BSM-119, BS-120, and BS-121) isolated from *Chelidonium majus* seeds and six *Pseudomonas* strains isolated from *Cannabis sativa* roots (PF-1, PPW-26, PFM-34, and PFM-48) and stems (PF-69 and PS-72) were reported. Whole genome sequencing of these strains was performed by Day Zero Diagnostics (MA, USA). Library preparation, DNA fragmentation, and barcode attachment were performed using transposase-based tagmentation with the Rapid Barcoding Sequencing Kit (SQK-RBK114.96), followed by cleanup and size selection (≥150 bp) using solid-phase reversible immobilization (SPRI) beads. Long-read sequencing was performed using the PromethION-24 platform with the FLO-PRO114M flow cell, and quality was monitored by the MiniKNOW interface from Oxford Nanopore Technologies (ONT). Base calling and demultiplexing were conducted using Guppy v.6.5.7 (ONT) (9). Quality control involved down-sampling reads to 500–1,500 Mb per sample, followed by trimming and filtering with Fastp v.0.23.2. (10). Trimmed and filtered reads were assembled *de novo* with Flye v.2.9.2 (11) using the --nano-hq and—meta parameters. Assembly quality was assessed with QUAST, excluding short contigs (<500 bp) and those with low depth (<5×). The resulting assemblies were annotated using NCBI’s PGAP. Taxonomic assignments were based on NCBI annotation and sequence similarity, with organisms reported at the genus level and, where supported, at the species level. Strain typing was conducted *in silico* using the PubMLST database. Detailed genome assembly statistics are provided in Table 1. Default parameters were used for all programs unless otherwise specified.

**TABLE 1:** Statistical genomic information of the 13 bacterial isolates

Sample ID	Genomic species result	GenBank accession	SRA accession	Total reads	Genome total length (bp)	Genome coverage (x)	Contigs	% Q20 Mbs	N50 value	GC content (%)	Bases	CDS	rRNA	tRNA	tmRNA
PF-1	*Pseudomonas fluorescens group sp*.	JBTMNG000000000	SRR37415448	123,485	7,026,999	132.3	4	90.1	3,694,186	59.1	7,026,999	6,504	22	71	1
PPW-26	*Pseudomonas wadenswilerensis*	JBTMNF000000000	SRR37415447	146,872	6,897,095	165.3	1	90.7	6,897,095	64.5	6,897,095	5,891	19	76	1
PFM-34	*Pseudomonas mohnii*	JBTMNE000000000	SRR37415444	171,873	6,646,982	221.2	1	90.3	6,646,982	59.5	6,646,982	6,024	22	70	1
PFM-48	*Pseudomonas mohnii*	JBTMND000000000	SRR37415443	209,908	6,749,983	217.8	1	90.3	6,749,983	59.5	6,749,983	6,094	22	70	1
PF-69	*Pseudomonas fluorescens group sp*.	JBTMNC000000000	SRR37415442	204,898	6,742,723	218.0	3	90.6	6,655,611	59.7	6,742,723	6,173	23	71	1
PS-72	*Pseudomonas sichuanensis*	JBTMNB000000000	SRR37415441	185,957	5,961,881	246.6	1	90.7	5,961,881	64.0	5,961,881	5,332	22	79	1
BS-114	*Bacillus subtilis*	JBTMNA000000000	SRR37415440	261,973	4,171,346	354.8	1	90.9	4,171,346	43.8	4,171,346	4,123	30	86	1
BS-115	*Bacillus subtilis*	JBTMMZ000000000	SRR37415439	452,124	4,284,415	343.1	1	89.8	4,284,415	43.5	4,284,415	4,308	30	86	1
BS-116	*Bacillus subtilis*	JBTMMY000000000	SRR37415438	231,009	4,004,069	367.1	1	89.3	4,004,069	43.9	4,004,069	3,936	30	86	1
BS-118	*Bacillus subtilis*	JBTMMX000000000	SRR37415437	327,358	4,225,456	279.3	1	88.8	4,225,456	43.5	4,225,456	4,203	30	86	1
BSM-119	*Bacillus mojavensis*	JBTMMW000000000	SRR37415446	287,005	3,994,629	368.0	1	90.6	3,994,629	43.7	3,994,629	3,975	30	86	1
BS-120	*Bacillus subtilis*	JBTMMV000000000	SRR37415445	221,049	4,138,660	355.2	2	90.0	4,078,843	43.6	4,138,660	4,243	30	89	1
BS-121	*Bacillus subtilis*	JBTMMU000000000	SRR37747387	66,769	4,138,661	119.1	2	93.9	4,078,844	43.6	4,138,661	4,245	30	89	1

## Data Availability

The whole genome sequences of the 13 bacterial isolates were deposited in the NCBI GenBank as Bioproject PRJNA1379652. The GenBank accession numbers are listed in Table 1. The version described in this paper is the first version.

## References

[1] Marchut-Mikolajczyk O, Drożdżyński P, Pietrzyk D, Antczak T. 2018. Biosurfactant production and hydrocarbon degradation activity of endophytic bacteria isolated from Chelidonium majus L. Microb Cell Fact 17:171. doi:10.1186/s12934-018-1017-5 30390702 PMC6215600

[2] Buirs L, Punja ZK. 2025. Endophytes in Cannabis sativa: identifying and characterizing microbes with beneficial and detrimental effects on plant health. Plants (Basel) 14:1247. doi:10.3390/plants14081247 40284136 PMC12030312

[3] Comeau D, Balthazar C, Novinscak A, Bouhamdani N, Joly DL, Filion M. 2021. Interactions between Bacillus spp., Pseudomonas spp. and Cannabis sativa promote plant growth. Front Microbiol 12:2021. doi:10.3389/fmicb.2021.715758 PMC8488376 34616381

[4] Balthazar C, Novinscak A, Cantin G, Joly DL, Filion M. 2022. Biocontrol activity of Bacillus spp. and Pseudomonas spp. against Botrytis cinerea and other Cannabis fungal pathogens. Phytopathology 112:549–560. doi:10.1094/PHYTO-03-21-0128-R 34293909

[5] Abdelshafy Mohamad OA, Ma J-B, Liu Y-H, Zhang D, Hua S, Bhute S, Hedlund BP, Li W-J, Li L. 2020. Beneficial endophytic bacterial populations associated with medicinal plant Thymus vulgaris alleviate salt stress and confer resistance to Fusarium oxysporum Front Plant Sci 11:47. doi:10.3389/fpls.2020.00047 32117385 PMC7033553

[6] Wu W, Chen W, Liu S, Wu J, Zhu Y, Qin L, Zhu B. 2021. Beneficial relationships between endophytic bacteria and medicinal plants. Front Plant Sci 12:646146. doi:10.3389/fpls.2021.646146 33968103 PMC8100581

[7] Khadka G, Shetty KG, Annamalai T, Tse-Dinh Y-C, Jayachandran K. 2024. Characterization and antimicrobial activity of endophytic fungi from medicinal plant Agave americana. Lett Appl Microbiol 77:ovae025. doi:10.1093/lambio/ovae025 38467396

[8] Gagne-Bourgue F, Aliferis KA, Seguin P, Rani M, Samson R, Jabaji S. 2013. Isolation and characterization of indigenous endophytic bacteria associated with leaves of switchgrass (Panicum virgatum L.) cultivars. J Appl Microbiol 114:836–853. doi:10.1111/jam.12088 23190162

[9] Wick RR, Judd LM, Holt KE. 2019. Performance of neural network basecalling tools for Oxford Nanopore sequencing. Genome Biol 20:129. doi:10.1186/s13059-019-1727-y 31234903 PMC6591954

[10] Chen S, Zhou Y, Chen Y, Gu J. 2018. Fastp: an ultra-fast all-in-one FASTQ preprocessor. Bioinformatics 34:i884–i890. doi:10.1093/bioinformatics/bty560 30423086 PMC6129281

[11] Kolmogorov M, Bickhart DM, Behsaz B, Gurevich A, Rayko M, Shin SB, Kuhn K, Yuan J, Polevikov E, Smith TPL, Pevzner PA. 2020. metaFlye: scalable long-read metagenome assembly using repeat graphs. Nat Methods 17:1103–1110. doi:10.1038/s41592-020-00971-x 33020656 PMC10699202

